# Identification of a novel mutation in the factor VIII gene causing severe haemophilia A

**DOI:** 10.1186/s12878-018-0113-4

**Published:** 2018-07-31

**Authors:** S. K. Nissen, A. L. Laursen, L. H. Poulsen, T. H. Mogensen

**Affiliations:** 1 Department of Infectious Diseases, Aarhus University Hospital, Aarhus, Denmark; 2 Department of Biomedicine, Aarhus University, Aarhus, Denmark; 3 Centre for Haemophilia and Thrombosis, Department of Clinical Biochemistry, Aarhus University Hospital, Aarhus, Denmark

**Keywords:** Haemophilia, Factor 8 gene mutation, Whole exome sequencing

## Abstract

**Background:**

Deficiency in coagulation factor VIII encoded by *F8* results in the X-linked recessive bleeding disorder haemophilia A (HEMA). Here we describe the identification of a novel variant in the factor VIII gene, *F8*, in an adult male patient with severe haemophilia A.

**Case presentation:**

The patient was diagnosed in early childhood and subsequently co-infected with Hepatitis C and HIV acquired during early blood transfusion for haemophilia in the 1980ies. The identified *F8* deletion, c.5411_5413delTCT, p.F1804del lies within a conserved part of the molecule, is predicted by bioinformatic software to be deleterious by the loss of Phenylalanine, and has not been previously described in any database.

**Conclusion:**

This novel *F8* deletion as a cause of haemophilia A did not result in generation of inhibitory antibodies to Factor VIII treatment and may have impact on (prenatal) diagnosis, genetic counselling, and treatment decisions in the affected family as well as in other families diagnosed with this *F8* mutation. Finally, this novel mutation should be included in the panel of known genetic variants in *F8* when searching for the genetic etiology in patients suspected of HEMA.

**Electronic supplementary material:**

The online version of this article (10.1186/s12878-018-0113-4) contains supplementary material, which is available to authorized users.

## Background

**Fig. 1:**
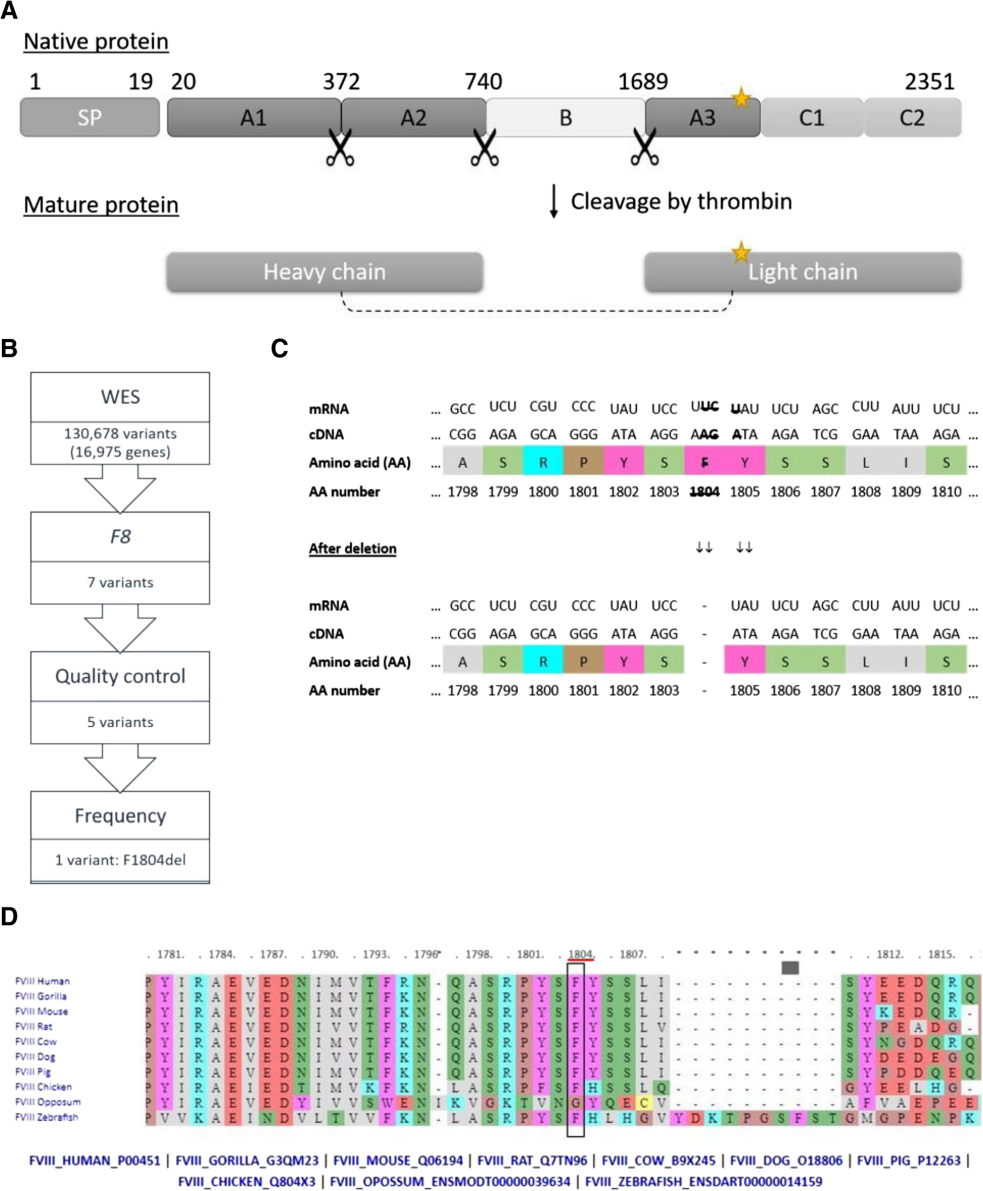
**a** Structure of novel and processed factor VIII protein. Signalling peptide (SP), novel mutation marked by star. **b** Identification of relevant variant by several filtering steps. **c** Section of the *F8* gene, mRNA and factor VIII amino acid (AA) sequence with affected bases and AA high-lighted showing the resulting deletion in the Factor VIII molecule. **d** AA position examined for conservation in human, gorilla, mouse, rat, cow, dog, pig, chicken, oppossum, and zebrafish F8 using the publicly available multiple-sequence alignment (http://www.factorviii-db.org/sequence.html.php)

Deficiency in coagulation factor VIII encoded by *F8* results in the X-linked recessive bleeding disorder haemophilia A (HEMA). HEMA occurs in 1:5.000–10.000 males with approximately one third of the cases being spontaneous mutations. The more than 180 kb long *F8* gene consists of 26 exons encoding a 2351 amino acids (AA) long precursor protein. The native protein consists of six domains: A1-A2-B-A3-C1-C2 and is cleaved into a heavy chain and a light chain structure by thrombin (Fig. 1a) [1, 2]. Several different mutations can lead to factor VIII deficiency. Whereas large intron inversions are responsible for almost half of the HEMA cases, deletions, duplications, and point mutations may also cause the disease [3–5]. HEMA is a diverse disease with a mild, moderate, and severe form with ≥5%, 2–5%, and ≤ 1% remaining protein activity, respectively [1]. Knowledge on the severity caused by each specific mutation can be important during prenatal testing for management of pregnancy and delivery [6].

## Case presentation

A 68-year-old male was diagnosed with severe HEMA in early childhood, with less than 0.001% factor VIII activity. The brother of the proband also suffered from severe HEMA, thus the mother must have been carrier of the causative mutation. The children of the proband were male, and consequently, in this part of the family the mutation has not been passed on. Due to the distant past of the diagnosis, no genetic tests had been performed to identify the causative mutation. Around 30% of patients with severe HEMA develop inhibitors during their treatment with factor VIII, especially patients with large deletions and intron inversions. Thus, genetic factors can influence inhibitor development, and different treatment approaches are chosen according to risk of inhibitor development [7]. However, the proband never developed factor VIII inhibitors, possibly suggesting a smaller and less frequent mutation in *F8* than the large intron inversion. Following blood transfusion, the proband was tested positive for HIV-1 and hepatitis C virus in the late 1980s and early 1990s, respectively. The patient was cured for his Hepatitis C infection, but never received any treatment for his HIV-1 infection, since he remained with normal CD4 T cell count over time and was considered an HIV long-term non-progressor (LTNP).

To identify the HEMA causative mutation (as well as possible mutations explanatory for his HIV LTNP phenotype), a blood sample was drawn in EDTA tubes (FLUKA), and peripheral blood mononuclear cells (PBMCs) were isolated over ficoll gradient (GE-healthcare). Integrating HIV DNA in CD4 T cells might result in false positive (somatic mosaic) mutations, or disturb the quality of sequencing. Therefore, CD4 T cells were depleted by magnetic purification (miltenyi biotec). DNA from non-CD4 T cells was purified using allprep DNA/RNA mini kit (Qiagen). Whole exome sequencing (WES) was performed employing Kapa HTP Library preparation and Nimblegen SeqCap EZ MedExome Plus kit and analysed using Nextseq v2 chemistry (2 × 150 bp). SNPs were called relative to hg19. Variant call files (VCF) were uploaded to Ingenuity Variant Analysis (IVA, Qiagen) and variants were compared to population frequencies of variants in the Allele Frequency Community (AFC) database and to frequencies in the 1000 Geneomes project. One hundred thirty thousand six hundred eighty-seven variants were identified in 16,957 genes in the patient, of which seven were located in the *F8* gene. Two variants did not pass quality control, thus five variants could be possibly causative (see Table 1). Four of the remaining variants had an allele frequency much higher than the disease frequency and were therefore judged as being irrelevant. Therefore, one variant (c.5411_5412delTCT, p.F1804del) remained a potential cause of disease (Fig. 1b). The mutation was verified in the raw BAM file (Additional file 1: Figure S1). Ingenuity did not provide any dbSNP ID or frequency for this variant, which is thus denoted as novel. Moreover, the variant was not reported in Coagulation Factor Variant Databases EAHAD.CFDB (https://databases.lovd.nl/shared/variants/*F8*), which provides all 5418 known transcript variants in the *F8* gene, confirming that the c.5411_5413delTCT, p.F1804del must indeed be novel.

**Table 1:** Identified genetic variants passing quality control

Transcript Variant	Protein Variant	Gene Region	Variant Type	Activity	Classification	Impact	CADD Score	dbSNP ID	AFC Freq.	1000 Genomes Freq.
c.6115 + 103 T > C		Intronic	SNV	normal	Benign		< 10	4,074,307	22.442	44.079
c.5998 + 91 T > A		Intronic	SNV	normal	Benign		< 10	4,898,352	22.653	44.132
c.5411_5413delTCT	p.F1804del	Exonic	Deletion	loss	Likely Pathogenic	in-frame	*			
c.3780C > G	p.D1260E	Exonic	SNV	gain	Benign	missense	< 10	1,800,291	18.924	25.642
c.1010-27G > A		Intronic	SNV	normal	Benign		< 10	7,058,826	11.328	7.735

Transcript ID NM_000132.3, chromosome X, cytoband q28, gene F8. * CADD score can not be estimated for deletions

*Freq* frequency, *CADD* combined annotation dependent depletion

## Discussion and conclusion

The three deleted bases in *F8* are distributed with two located in the end of F1804-codon and one in the beginning of Y1805-codon. The remaining bases from the two codons will fuse as new Tyr codon (see Fig. 1c). Thus, the three base deletion results in an in-frame p.F1804 deletion located in exon 16 in the A3 domain/light chain structure (Fig. 1c). This functional domain is highly conserved throughout evolution (Fig. 1d), stressing the importance of these specific AA in this area for a functional protein. Consequently, Ingenuity software predicts this deletion to be “likely pathogenic”, since it is 1) located in a critical and well-established domain without benign variation; 2) absent from controls; 3) and the protein length change in a non-repeat region. The F1804del variant was predicted to result in loss of function of the protein, whereas the more common variants were all predicted to have normal function or gain of function with low CADD score, thus interpreted as benign. Furthermore, a natural variant affecting the neighbouring AA (S1803Y, dbSNP:rs137852444) results in severe HEMA [8], and two natural variants affecting the same AA by substitution instead of deletion also result in severe HEMA, further supporting a disease causing role of the identified mutation. Finally, intron inversions responsible for the majority of HEMA cases were not identified in this patient. Taken together, these data indicate that c.5411_5413delTCT/p.F1804del is the causative mutation in the index patient.

Here we describe the identification of a novel deletion, p.F1804del in *F8* causing severe HEMA. Importantly, this novel deletion mutant did not result in generation of inhibitory antibodies to Factor VIIII treatment. The results from the present study demonstrate that WES can be used as a tool for identification of rare non-intron inversion mutations in *F8*, where PCR and denaturing gradient gel electrophoresis (DGGE) have been found inadequate to detect the HEMA causative mutation. Sanger sequencing may be used to examine for this variant in potential female carriers in the family of the index patient or for future prenatal diagnosis in the family. The identification of this specific *F8* deletion in an individual may have impact on genetic counselling, treatment decisions, and prediction of prognosis. Based on the present report, the *F8* p.F1804del is expected to predict a severe clinical phenotype of HEMA but possibly without generation of inhibitory antibodies to Factor VIII treatment. Finally, this novel mutation should be included in the panel of known genetic variants in *F8* when searching for the specific genetic etiology in patients suspected of HEMA.

## Additional file

Additional file 1: **Figure S1.** BAM file aligned to the *F8* gene in the UCSC genome browser. The three base deletion is visualized with absent alignment of library cDNA in both reading directions (red and blue). (DOCX 1688 kb)

## Abbreviations

AA: Amino acid
HEMA: Haemophilia A
LTNP: Long-term non-progressor
SNP: Single nucleotide polymorphism
WES: Whole exome sequencing
