# Supplementary figures and images for: Identification of a novel mutation in the factor VIII gene causing severe haemophilia A
Source: BMC Hematol. 2018 Jul 31;18:17. doi: 10.1186/s12878-018-0113-4 (PMC6069878; doi:10.1186/s12878-018-0113-4)

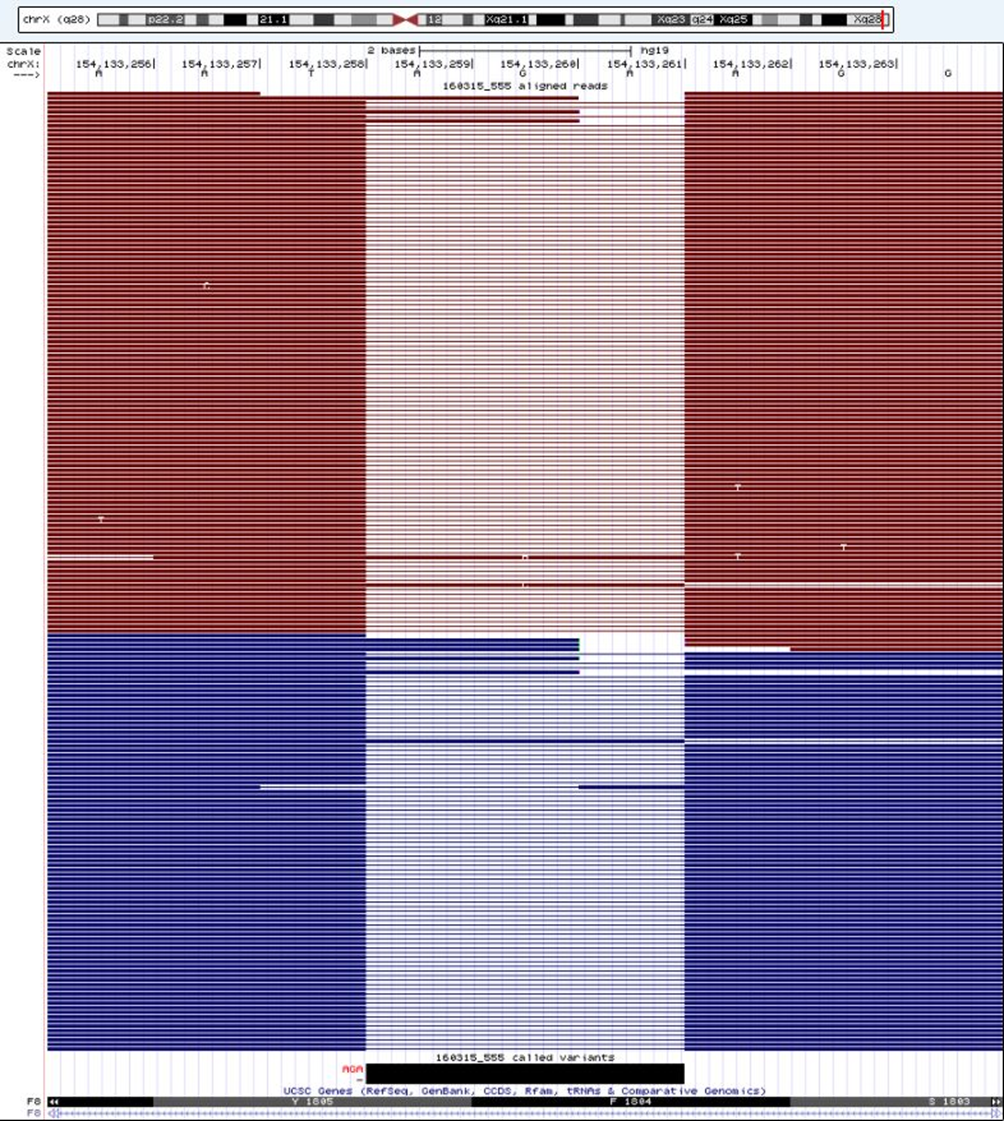

Supplement: Supplementary file 1 — Figure S1. BAM file aligned to the F8 gene in the UCSC genome browser. The three base deletion is visualized with absent alignment of library cDNA in both reading directions (red and blue). (DOCX 1688 kb) [file 12878_2018_113_MOESM1_ESM.docx]
